# *Drosophila melanogaster* Limostatin and Its Human Ortholog Promote West Nile Virus Infection

**DOI:** 10.3390/insects15060446

**Published:** 2024-06-12

**Authors:** Ezra B. Mead, Miyoung Lee, Chasity E. Trammell, Alan G. Goodman

**Affiliations:** 1 School of Molecular Biosciences, College of Veterinary Medicine, Washington State University, Pullman, WA 99164, USA; 2 Department of Microbiology, Immunology, and Pathology, College of Veterinary Medicine and Biomedical Sciences, Colorado State University, Fort Collins, CO 80523, USA; 3 Paul G. Allen School of Global Health, College of Veterinary Medicine, Washington State University, Pullman, WA 99164, USA

**Keywords:** fruit fly, mosquito, vector, insulin, neuromedin, flavivirus

## Abstract

**Simple Summary:**

Insect-borne viruses, such as those of the Flaviviridae family, pose a serious risk to global health. WNV, a mosquito-borne flavivirus, is transmitted primarily by the *Culex* mosquito. Despite the increasing exposure of populations to mosquito-borne flaviviruses and the expanding range of the vector mosquito, there are limited resources available to prevent or treat flavivirus infections. Using the model organism *Drosophila melanogaster*, commonly known as the fruit fly, we previously found that insulin signaling reduces WNV infection. We translated these finding to mosquitoes and human cells and showed similar mechanisms of insulin-mediated antiviral activity. However, insect and mammalian hormones can regulate insulin signaling. Specifically, decretin hormones suppress insulin secretion, especially during periods of starvation and low glucose intake. In this study, we show that the insect decretin, Limostatin, and its mammalian ortholog, Neuromedin U, can promote WNV infection. These results suggest that the inhibition of decretin signaling may be a novel therapeutic target to control WNV infection.

**Abstract:**

The arbovirus West Nile virus (WNV) is a danger to global health. Spread primarily by mosquitoes, WNV causes about 2000 cases per year in the United States. The natural mosquito immune response controls viral replication so that the host survives but can still transmit the virus. Using the genetically malleable *Drosophila melanogaster* model, we previously dissected innate immune pathways used to control WNV infection. Specifically, we showed that insulin/IGF-1 signaling (IIS) activates a JAK/STAT-mediated immune response that reduces WNV. However, how factors that regulate IIS in insects control infection has not been identified. *D. melanogaster Limostatin* (*Lst*) encodes a peptide hormone that suppresses insulin secretion. Its mammalian ortholog, Neuromedin U (NMU), is a peptide that regulates the production and secretion of insulin from pancreatic beta cells. In this study, we used *D. melanogaster* and human cell culture models to investigate the roles of these insulin regulators in immune signaling. We found that *D. melanogaster Lst* mutants, which have elevated insulin-like peptide expression, are less susceptible to WNV infection. Increased levels of insulin-like peptides in these flies result in upregulated JAK/STAT activity, leading to protection from infection. Treatment of human cells with the insulin regulator NMU results in increased WNV replication. Further investigation of methods to target Lst in mosquitoes or NMU in mammals can improve vector control methods and may lead to improved therapeutics for human and animal infection.

## 1. Introduction

The flavivirus WNV poses a global health threat [1] and has been present in the United States since it first made landfall in New York in 1999 [2]. WNV is spread by mosquitoes, primarily the mosquito species *Culex quinquefasciatus* [3]. Climate change is altering mosquito habitats, feeding activity, and seasonal patterns [4,5]. This allows mosquitoes, and the viruses they carry, to move into new areas, spreading those diseases further [6,7]. Symptoms and progression of WNV vary in humans due to genetic variation [8,9]. WNV causes symptoms in 20% of cases [10], which can include conditions like headache, weakness, and rash [11]. Furthermore, 1 in 150 cases may develop the more severe West Nile neuroinvasive disease, which can feature encephalitis [12], meningitis [3], neuronal cell death [13], and death.

*Drosophila melanogaster* is a useful model to study immunity and the control of viral infection in mosquito vectors like *Cx. quinquefasciatus*. There is wide genetic diversity between *D. melanogaster* and mosquitoes [14], but many components of the immune system are conserved [15]. The genetic malleability of *D. melanogaster* provides a tool to study specific mutations that affect insect immunity. Our lab previously used the Kunjin virus strain of WNV (WNV-Kun) to perform a genetic screen in *D. melanogaster* [16] due to its similarity to the 1999 New York strain [17] and the virulent Linage 1a strain [18,19,20]. We found that variants in the *Insulin receptor* (*InR*) gene rendered flies more susceptible to WNV-Kun.

Various signaling pathways have an impact on viral infections in *D. melanogaster* [21,22,23]. *D. melanogaster* and mosquitoes utilize similar antiviral response pathways, specifically RNAi and JAK/STAT, as primary means of protection against viral infection [15,24]. RNAi machinery degrades detected cytosolic viral nucleic acids. JAK/STAT induces antiviral cytokines to act upon viral-stimulated ligands. Insulin/insulin-like growth factor 1 (IIS) and MAPK/ERK pathways are also antiviral during infection in insects [16,25]. The insulin/IGF-1 signaling (IIS) pathway stimulates the activation of the JAK/STAT antiviral pathway [16], so the role of proteins that control IIS-mediated immunity in insects should be examined.

Decretins are hormones that suppress insulin production and secretion under starvation conditions [26]. Decretins exist in insects and mammals [26]. Hormonal systems for metabolic regulation, including insulin signaling, are largely conserved in mammals [27]. *D. melanogaster limostatin* (*Lst*) encodes a peptide hormone decretin, Lst, that suppresses insulin secretion [28]. A conserved 15-residue Lst polypeptide is produced in glucose-sensing enteroendocrine gut-associated cells. Lst production is suppressed by carbohydrate feeding. Limostatin deficiency leads to hyperinsulinemia, hypoglycemia, and excess fat storage [26]. Glucose-stimulated insulin secretion is regulated by different metabolic states due to feeding behavior. Circulating insulin is elevated during times of feeding, leading to increased nutrient storage. In starvation or low-nutrient conditions, insulin is decreased to signal nutrient mobilization [29]. Gut-associated hormones play a role in regulating insulin secretion in response to carbohydrate intake. *D. melanogaster* insulin-like peptides (ilps) produced in neuroendocrine cells regulate nutrient storage in the fly in response to elevated circulating glucose after food intake [26].

The mammalian ortholog of Lst is Neuromedin U (NMU). *CG9918* encodes the *D. melanogaster* Lst receptor, and its mammalian ortholog is the NMU receptor (NMUR). Knockdown of *CG9918* in insulin-producing cells (IPCs) decreases insulin secretion. This Lst receptor was identified as a G-coupled protein receptor (GPCR) in IPCs [26]. The GPCR NMUR1 is present in mammalian pancreatic beta cells [30], where stimulation by NMU leads to decreased insulin secretion [31,32]. Since our lab previously described the mechanism of insulin-mediated immune signaling during WNV infection, we next sought to investigate the role of the insulin regulators Lst and NMU in immunity to WNV infection. In this study, we show that *Lst* mutant *D. melanogaster* shows elevated expression of *insulin-like peptides* (*ilps)* and elevated expression of genes within the JAK/STAT pathway during infection. These mutants are less susceptible to WNV-Kun infection. Normal human fibroblasts expressing NMU receptor 1 (NMUR1) and treated with NMU-25 peptide show higher viral titer following infection than control cells. This research indicates that suppressors of insulin secretion have a direct impact on susceptibility to WNV-Kun infection. Understanding these immune pathways and factors of susceptibility will have a direct impact on improving therapeutics for diabetic individuals and patients with hypoglycemic symptoms.

## 2. Methods

### 2.1. Cell Culture and Virus Production

Normal human fibroblast 1 (NHF1, courtesy of Dr. John Wyrick) and Baby Hamster Kidney 21 (BHK21, ATCC) cells were cultured at 37 °C/5% CO_2_ in DMEM (ThermoFisher 11965-1118, Bothell, WA, USA) with 10% Fetal Bovine serum (Atlas EF-0500-A, Fort Collins, CO, USA) and 1× antibiotic–antimycotic (ThermoFisher 15240-062, Bothell, WA, USA). Cells were passaged every 3 days. West Nile virus–Kunjin (strain MRM16) was provided by R. Tesh, grown in Vero cells (ATCC), and purified through ultracentrifugation. WNV-Kun can be used in arthropod containment level 2 (ACL2) facilities [33,34]. All experiments with a specific virus type utilized the same stock.

### 2.2. Fly Mortality Study

First, 2–5-day-old female *D. melanogaster* were anesthetized with CO_2_ and injected intrathoracically with 23 nl of WNV-Kun at a dose of 200 PFU/fly. Mock infection was performed using PBS injection. For each independent experiment, 40 flies of each genotype, *y^1^w^1^* (Bloomington *Drosophila* Stock Center (BDSC) #1495) and y^1^w*; *Mi{y^+mDint2^ = MIC}Lst^MI06290^* (BDSC #60793), were infected with WNV-Kun or mock infected and kept on vials with standard cornmeal food (Genesee Scientific, Morrisville, NC, USA). Surviving flies were counted every 24 h for 30 days. Vials were changed every three days. Hazard ratios were calculated in the Survival Curve analysis program in Graphpad Prism ver. 9. A hazard ratio is an index of effect size and compares the rates of mortality over time between two survival curves [35]. Survival curves represent data from three replicate experiments combined together.

### 2.3. Virus Replication Assay

Virus replication in flies was measured using a standard plaque assay on BHK21 cells. First, 2–5-day-old female flies were infected with 23 nL of WNV-Kun at a dose of 2000 PFU/fly. Flies were kept on vials with standard cornmeal food, and vials were changed every three days. At 1, 5, and 10 days post-infection, three sets of five flies were collected from each genotype. Flies were homogenized in Phosphate-Buffered Saline (PBS) before virus titration. The homogenate was serially diluted in DMEM with 2% FBS and plated on a 12-well plate of BHK21 cells at 1.5 × 10^5^ cells/well. Plates were incubated for 2 h at 37 °C/5% CO_2_ and rocked every 15 min. Wells were overlayed with 4% low-melting-point agarose (Invitrogen 16520050, Waltham, MA, USA) for a final concentration of 0.75% agarose and 4% FBS in DMEM. Plates were incubated for four days at 37 °C/5% CO_2_ before visualization with 0.1% crystal violet (Fisher 548-62-9, Hampton, NH, USA).

### 2.4. Quantitative Reverse Transcriptase PCR

qRT-PCR was conducted to measure target mRNA expression levels in *D. melanogaster* or NHF1 cells. Groups of five flies per sample or 2 × 10^5^ NHF1 cells in a 12-well plate were lysed in Trizol reagent (ThermoFisher 15-596-026, Bothell, WA, USA). RNA was extracted using the Direct-Zol RNA Miniprep kit. DNA was removed with DNase I (Invitrogen 18068-015) and cDNA was prepared using the iScript cDNA Synthesis kit (Bio-Rad 170-8891, Hercules, CA, USA). Expressions of *ilp-2*, *ilp-3*, *ilp-5*, and *ilp-7* were measured using SYBR Green reagents (Fisher K0222, Hampton, NH, USA) and normalized to *rp49*. Flies infected with WNV-Kun in the manner described above were used to measure the expression of *vir-1* and *upd-3*, normalized to *rp49* expression. The PCR reaction included one cycle of denaturation at 95 °C for 10 min, followed by 50 cycles of denaturation at 95 °C for 15 s and extension at 60 °C for 1 min, using an Applied Biosystems 7500 Fast Real Time PCR System. ROX was used as an internal control. All primer sequences were previously published as follows: *ilp-2*, *ilp-3*, *ilp-5* [36]; *ilp-7* [37]; *vir-1* [38]; *upd-3* [16]; *rp49* [39].

### 2.5. Preparation of pcDNA3.1;NMUR1 Plasmid

pcDNA3.1;NMUR1 plasmid was a generous donation from the lab of Dr. Ching-Wei Luo. Plasmid was cloned through transfection into chemically competent *E. coli* cells and extracted for experimental use using the GeneJET plasmid miniprep kit (Fisher K0503, Hampton, NH, USA)

### 2.6. Transfection of Plasmids into Cells

pcDNA3.1;NMUR1 plasmid was transfected into NHF1 cells in a 12-well plate using a concentration of 1 µg of DNA in each well. Transfection was conducted by combining 2.5 μL of lipofectamine (ThermoFisher, 11668019, Bothell, WA, USA) and 125 μL of Optimem (ThermoFisher 31985062, Bothell, WA, USA) in a microcentrifuge tube and 1 µg DNA and 125 μL of Optimem in a separate tube and incubating at room temperature for 5 minutes before mixing and incubating for 20 min. pcDNA3.1+ vector was transfected into cells as an empty vector control.

### 2.7. Western Blot

Protein extracts were prepared by lysing adult flies or cells in RIPA buffer (25 mM of Tris-HCl (pH 7.6), 150 mM of NaCl, 1 mM of EDTA, 1% NP-40, 1% sodium deoxycholate, 0.1% SDS, 1 mM of Na_3_VO_4_, 1 mM of NaF, 0.1 mM of PMSF, 10 μM of aprotinin, 5 μg/mL of leupeptin, 1 μg/mL of pepstatin A, and 10 nM of DTT). Protein samples were diluted using 2x Laemmli loading buffer (Eco-tech LSB10x), mixed, and boiled for 5 min at 95 °C. Samples were analyzed through SDS/PAGE using a 10% acrylamide gel, followed by transfer onto a PVDF membrane (Sigma-Aldrich MABF213, St. Louis, MO, USA). Membranes were blocked with 5% non-fat dry milk (NFDM) in Tris-buffered saline (50 mM of Tris-HCl pH 7.5, 150 mM of NaCl) and 0.1% Tween-20 for 1 h at room temperature. NHF1 cells transfected with pcDNA3.1;NMUR1 or empty vector were lysed at 24, 48, and 72 h post-transfection in RIPA and immunoprecipitated with an antibody recognizing FLAG or actin in 5% NFDM in Tris-buffered saline and 0.1% Tween-20 at 4 °C overnight. Primary antibody labeling was performed with anti-Akt (1:2000) (Cell Signaling, 4691), anti-phospho-Akt (1:1000) (Cell Signaling, 4060), anti-FLAG (1:1000) (Sigma-Aldrich, F1804), or anti-actin (1:10,000) (Sigma-Aldrich, A5441) antibodies. Secondary antibody labeling was performed with anti-mouse or -rabbit IgG HRP conjugate antibody (1:10,000) (Promega W4021, W4011, Madison, WI, USA) by incubating membranes for 2 h in 1% NFDM in Tris-buffered saline and 0.1% Tween-20 at 4 °C.

### 2.8. Infection of NMUR1-Expressing Cells

NFH1 cells were cultured at a concentration of 2.0 × 10^5^ cells/well in a 12-well plate and transfected with either pcDNA3.1;NMUR1 or empty vector plasmid. Six hours later, three biological replicates were supplemented with 100 nM of NMU-25 peptide (Aapptec P002126, Louisville, KY, USA). Then, 24 h later, cells were infected at a dose of 0.01 MOI PFU/cell with WNV-Kun. At 72 h post-infection, cell culture supernatant was collected for use on a viral plaque assay.

### 2.9. Quantification and Statistical Analyses

**Figure 1:**
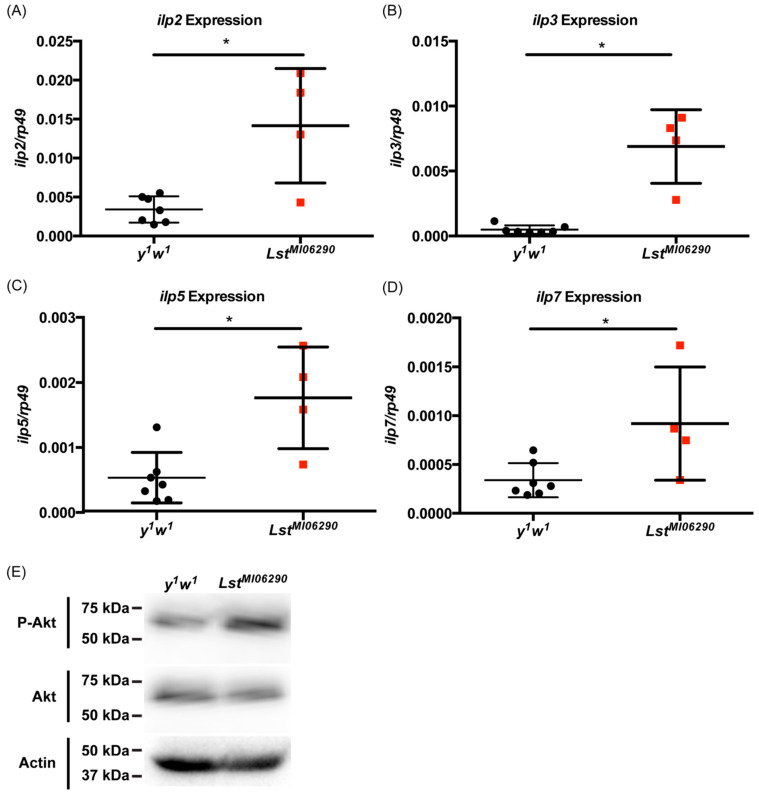
*Lst^MI06290^* mutant *D. melanogaster* are hyperinsulinemic and exhibit increased Akt phosphorylation. *Ilp* expression and Akt phosphorylation were measured in adult (2–5-day-old) female flies (N = 5 flies per biological replicate). Gene expression was normalized to expression of the housekeeping gene *rp49*. (**A**) *ilp2*, (**B**) *ilp3*, (**C**) *ilp5*, and (**D**) *ilp7* expression in adult *Lst^MI06290^* flies compared to controls (*y^1^w^1^*). (**E**) Fly lysates were subjected to western blot for phospho-Akt, total Akt, and actin. Original gels are provided as Supplementary Figure S1. Results are representative of three independent experiments. * *p* < 0.05 (unpaired *t*-test).

**Figure 2:**
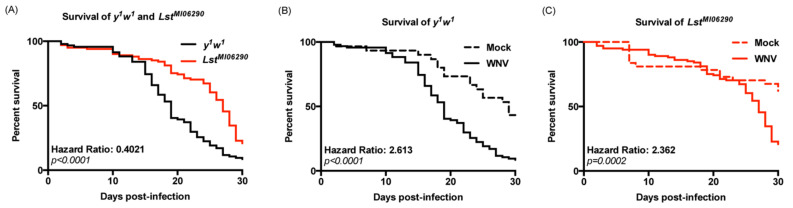
*Lst^MI06290^* mutant flies are less susceptible to WNV infection. Adult (2–5-day-old, N = 40 flies per experiment) *Lst^MI06290^* mutant and *y^1^w^1^* control flies were mock-infected or infected with WNV, and survival was monitored for 30 days. (**A**) Survival of WNV-infected flies of *Lst^MI06290^* and control genotypes. (**B**) Survival of control fly genotype *y^1^w^1^* infected with PBS mock or WNV. (**C**) Survival of *Lst^MI06290^* flies infected with PBS mock or WNV. Each survival curve represents three independent experiments (Table 1) that were combined for a final survival curve and statistical analyses.

**Figure 3:**
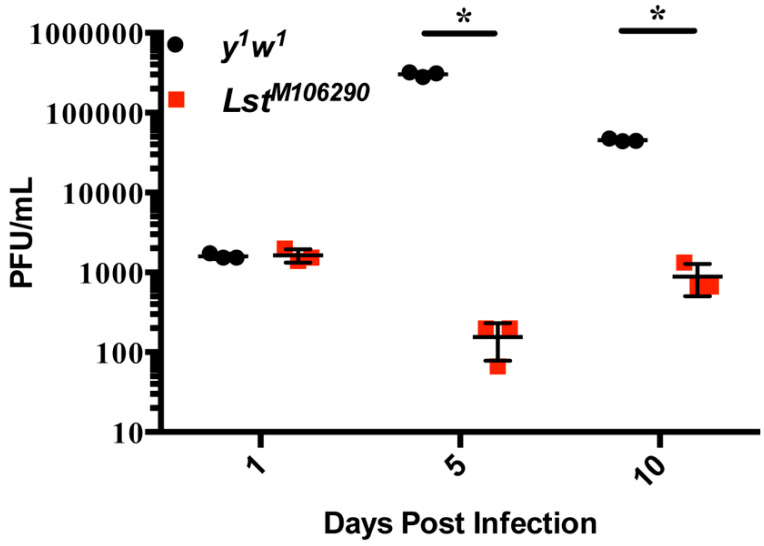
WNV replicates less in *Lst^MI06290^* mutant flies. Adult (2–5-day-old) female *Lst^MI06290^* or control flies (N = 5 flies per biological replicate) were mock-infected or infected with WNV (2000 PFU/fly) for 1, 5, and 10 days. Viral titer was determined via plaque assay. Results are representative of three independent experiments. * *p* < 0.0001 (one-way ANOVA).

**Figure 4:**
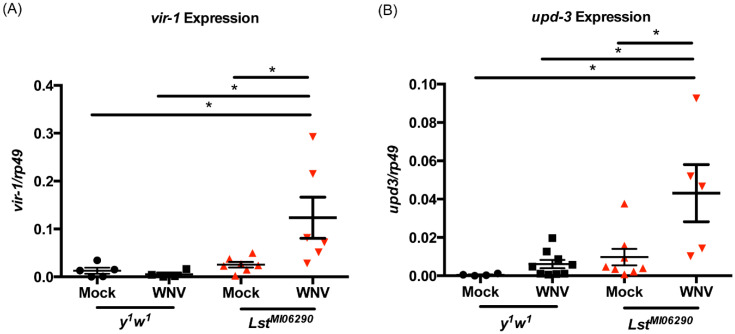
Expression of *vir-1* and *upd-3* genes is upregulated in WNV-infected *Lst^MI06290^ flies.* Adult (2–5-day-old) female *Lst^MI06290^* flies and control flies (N = 5 flies per biological replicate) were mock-infected or infected with WNV. Five days post-infection, flies were collected to measure expression of (**A**) *vir-1* and (**B**) *upd-3* using qRT-PCR. *Vir-1* and *upd-3* expression was normalized to *rp49* expression. Results are representative of four independent experiments. * *p* < 0.05 (one-way ANOVA).

**Figure 5:**
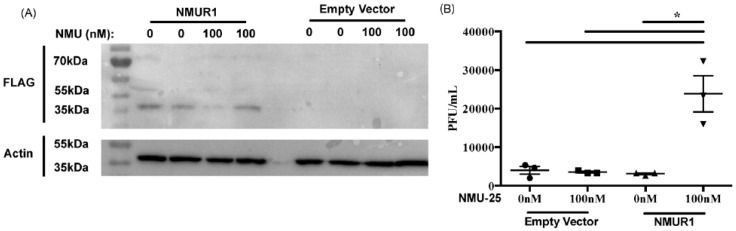
Normal human fibroblasts with an active NMUR1 pathway are more susceptible to WNV infection. NHF1 cells (N = 3 wells of cells) were transfected with an NMUR1-expressing plasmid or an empty vector control and treated with either 0 nM or 100 nM of NMU-25 peptide 6 h post-transfection. Then, 24 h later, cells were infected with WNV-Kun (MOI 0.01 PFU/cell). At 72 h post-infection, cells were lysed, and cell culture supernatant was collected. (**A**) Cell lysate was subjected to western blot for FLAG-tagged NMUR1 and actin. Original gels are provided as supplementary figures. (**B**) Supernatant used in a standard plaque assay to measure viral titer. Results are representative of three independent experiments. * *p* < 0.0001 (one-way ANOVA).

Results shown are representative of at least three independent experiments. Data points in dot plots represent a biological replicate of a pool of five flies (Figures 1, 3, and 4) or an individual well of cells (Figure 5). Statistical analyses were completed using GraphPad Prism. Two-tailed unpaired *t*-tests assuming unequal variance were utilized to compare normally distributed pairwise quantitative data. One-way analysis of variance with Tukey’s correction for multiple comparisons was used to compare multivariate data. Statistical tests were performed for each independent experiment to verify the robustness of the results. All error bars represent the standard error of the mean. Survival curves (Figure 2) represent three replicate experiments per condition pooled together and analyzed using the log-rank (Mantel-Cox) test using GraphPad Prism to determine *p* values between infected genotypes.

## 3. Results

### 3.1. Hyperinsulinemic D. melanogaster Models Are Less Susceptible to WNV Infection

*Lst* suppresses the release of *ilps* from insulin-producing cells, and mutation of *lst* causes increased expression of *ilps* [26]. Our lab has previously described how *ilp* signaling mediates the JAK-STAT innate immune response to WNV. We sought to determine if mutation of *Lst* would cause a hyperinsulinemic phenotype. *D. melanogaster* encodes for 8 *ilps* [40]. *D. melanogaster ilp7* is the most conserved to a mosquito *ilp* [41,42,43], while *D. melanogaster ilps 1–5* are most conserved to human and mouse insulin peptides [44]. Lst normally suppresses ilp production and secretion [26]. The *Lst^MI06290^* mutant fly line contains a mutation through the insertion of a transposable element to the *Lst* gene [45]. We measured the expression of insulin-like peptides in uninfected *Lst^MI06290^* and *y^1^w^1^* flies and show that *ilp-2*, *ilp-3*, *ilp-5*, and *ilp-7* are significantly upregulated in the *Lst^MI06290^* mutant fly line (Figure 1A–D). The *Lst^MI06290^* mutant fly line expresses ilps at higher levels than control flies. Because insulin signaling in flies results in increased Akt phosphorylation [16], and an increase in *ilp* gene levels does not necessarily correlate with protein levels [46], we next examined if *Lst^MI06290^* mutant flies showed increased insulin signaling via Akt activation. Indeed, immunoblotting confirms that *Lst^MI06290^* mutant flies exhibit increased Akt phosphorylation compared to control flies (Figure 1E). Together, these results support the use of the *Lst^MI06290^* fly line to model hyperinsulinemia, as the *Lst^MI06290^* flies display phenotypes similar to that described in Alfa et al [26]. We can then use this model to test how hyperinsulinemia affects WNV infection.

We next examined the rate of mortality to WNV-Kun infection in the hyperinsulinemic *Lst^MI06290^* mutant fly compared to *y^1^w^1^* control flies. Over a 30-day period following WNV-Kun infection via intrathoracic injection, *Lst^MI06290^* flies succumbed to infection at a significantly slower rate than control flies (Figure 2A). Comparison of survival was determined using a hazard ratio. A hazard ratio compares the rates of mortality over time between two survival curves, and it is an index of effect size [35]. Both the *Lst^MI06290^* line and *y^1^w^1^* lines showed higher rates of mortality during infection with WNV-Kun than during a mock infection. The *y^1^w^1^* line exhibits a 2.613 hazard ratio when comparing infection with WNV-Kun to mock infection (Figure 2B), while the *Lst^MI06290^* line exhibits a 2.362 hazard ratio when infected with WNV-Kun compared to mock infection (Figure 2C). This indicates higher survivability of the *Lst^MI06290^* line to WNV-Kun compared to mock infection. When comparing WNV-Kun infection of the *Lst^MI06290^* mutant line to the *y^1^w^1^* control, there is a 0.4021 hazard ratio, indicating the *Lst^MI06290^* line died at a slower rate than the control (Figure 2A). The results from each independent experiment that were combined for presentation in Figure 2 are presented in Table 1. In summary, the loss of the insulin-regulating Lst peptide led to lower mortality following WNV-Kun infection in hyperinsulinemic flies.

Because control flies exhibited increased susceptibility to WNV infection compared to *Lst^MI06290^* mutant flies, we next examined if WNV replication was correlated to this difference in mortality. Following infection with WNV-Kun, *y^1^w^1^* and *Lst^MI06290^* mutant flies were collected for use on a standard plaque assay using BHK21 cells. *Lst^MI06290^* flies showed significantly less viral replication at 5 and 10 days post-infection than the control flies (Figure 3).

### 3.2. JAK/STAT Expression Is Upregulated in Lst Mutant D. melanogaster

In *D. melanogaster*, the RNAi and JAK/STAT pathways are involved in the response to various viruses, including WNV [47,48]. The RNAi and JAK/STAT pathways are also used in response to WNV [49] in the mosquito species *A. aegypti* [50] and *C. quinquefasciatus* [51,52]. Insulin-mediated immunity is also involved in the response to WNV infection in *D. melanogaster* [16]. This pathway is activated through the binding of ilps to the insulin receptor (InR). The InR is expressed in the midgut of both *D. melanogaster* and mosquitoes [53,54,55]. The IIS pathway impacts both the RNAi and JAK/STAT pathways. Following the phosphorylation of Akt, the transcription factor FoxO is localized outside of the nucleus, decreasing the production of Dicer-2 and Argonaute-2, proteins used in the RNAi complex [56]. The activation of Akt also results in the phosphorylation cascade of the MAPK/ERK pathway, which produces upd2 and upd3 [16], the proteins that activate the antiviral JAK/STAT pathway. Activation of the JAK/STAT antiviral pathway leads to the expression of antiviral effectors, including *vir-1* and *TotM* [16,57].

To determine if the JAK/STAT pathway and its downstream cytokines were more highly activated in *Lst* mutants, expressions of the genes *upd-3* and *vir-1* were measured in mock- or WNV-infected *Lst^MI06290^* mutant and *y^1^w^1^* control flies. Expression of *upd-1* and *vir-1* was significantly higher in infected *Lst^MI06290^* mutant flies compared to mock-infected and control flies (Figure 4). Interestingly, we did not observe induction of *upd-3* or *vir-1* during WNV infection in control flies. This may be due to the low dose of infection or the timing of sample collection. Additionally, WNV infection alone did not induce *upd-3* or *vir-1* in *Drosophila* S2 cells [16]. Nevertheless, these results indicate that the hyperinsulinemic *Lst^MI06290^* mutant fly shows upregulation of the JAK/STAT immune signaling pathway during WNV infection.

### 3.3. The Human Ortholog of Lst Promotes WNV Infection in Human Fibroblasts

The human ortholog for limostatin is Neuromedin U, a peptide involved in feeding behavior, insulin regulation, and promoting the expression of inflammatory cytokines in adaptive immune cells [58]. The expression of NMUR1 (NMU receptor 1) in HEK-293T cells has been used to demonstrate that NMU signaling suppresses proliferation of SKOV-3 ovarian cancer cells [59]. In our experiments, we used the NHF1 cell line, as fibroblasts are infected when a WNV-infected mosquito takes a human bloodmeal [60], and these cells express IGF-1 receptor (IGF-1R) and secrete IGF-1 [61,62]. To model the regulation of WNV infection by NMU in NHF1 cells, we first expressed NMUR1 in NHF1 cells (Figure 5A).

NHF1 cells expressing NMUR1 were treated with NMU-25 peptide to activate NMUR1. These cells were then infected with WNV-Kun, and cell culture supernatant was used for a standard plaque assay with BHK21 cells to measure viral titer. NFH1 cells expressing an active NMUR1 pathway and treated with 100 nM of NMU-25 peptide showed a significantly higher viral titer three days post-infection compared to cells transfected with the empty vector control plasmid or NMUR1-expressing cells not treated with NMU-25 peptide (Figure 5B). Thus, like flies expressing *Lst* compared to mutant flies, human cells expressing the NMU pathway and treated with the insulin regulator NMU exhibited increased WNV replication.

In summary, insulin plays a key role in the immune response to WNV through the activation of the anti-viral JAK/STAT pathway [16]. We have shown that flies that are hyperinsulinemic due to a mutation in the decretin hormone-producing gene *Lst* are less susceptible to infection and show upregulation of the JAK/STAT pathway during infection. When the insulin-secretion-suppressing human ortholog of *Lst*, NMU, is supplemented to human fibroblasts expressing NMUR1, the cells show a higher viral titer after infection. These results indicate insulin-suppressing hormones to be important in the innate immune response to WNV infection in insect and mammalian models.

## 4. Discussion

Insulin-mediated immune signaling is important in *D. melanogaster*, the mosquito, and the human cell response to WNV. Insulin has been implicated in insect immune signaling [63]. For example, fly mutants of the InR substrate *chico* have increased resistance to bacterial infection [64]. *Thor*, a gene involved in *D. melanogaster* host immune defense [65], is upregulated two-fold in infected *chico* mutants due to activation by higher FoxO activity induced by decreased insulin signaling [66]. FoxO is a transcription factor in the RNAi antiviral signaling cascade that is downregulated in the presence of insulin [16] and is also known to induce antimicrobial peptide genes in the fly fat body [66]. In the *D. melanogaster* antiviral response, insulin feeding activates the MAPK/ERK to restrict viral infection [25]. Insulin priming activates the JAK/STAT antiviral pathway in *D. melanogaster* and mosquito cells [26]. Feeding insulin to mosquitoes suppresses the RNAi pathway and activates the JAK/STAT antiviral pathway to suppress replication of another flavivirus, Zika virus [67]. Here, we demonstrate that *D. melanogaster* with a mutation in the insulin suppression gene *Lst* is hyperinsulinemic and shows upregulated expression of genes within the antiviral JAK/STAT pathway during infection. It is likely that upregulated *ilps* in the *Lst^MI06290^* mutant fly leads to stimulation of the IIS/IGF signaling pathway, resulting in upregulated expression of the JAK/STAT immune signaling pathway. It is possible that the upregulation of this antiviral pathway due to the loss of the insulin-regulator Lst contributes to decreased susceptibility to infection in *Lst^MI06290^* mutant flies.

InR and IGF1-R signaling modulates downstream immune cell processes in human innate immune cells. InR and IGF1-R are expressed in monocytes and macrophages [68,69]. Insulin treatment of in vitro human monocytes induces the production of pro-inflammatory cytokines IL-6 and TNFα [70]. Regulation of insulin signaling may influence these InR/IGF1R-dependent innate immune responses. Lst is a putative ortholog for the human-insulin-secretion-suppressing peptide NMU. NMU is known to have a role in the adaptive immune response in human cells. NMU signals a cascade activating MAK1/2, ERK1/2, and P13K genes within pro-inflammatory pathways in adaptive immune cells [58]. NMUR is present in T cells, natural killer cells, and eosinophils. Activation of the NMU pathway via NMUR1 induces the release of pro-inflammatory interleukins [31]. This inflammatory response is known to be antiviral. NMUR1 is upregulated in G2 innate lymphoid cells, and NMU is upregulated during Acute Respiratory Syncytial virus infection, activating the release of antiviral interleukins IL-33 and IL-25 [71]. It is known that another neuromedin peptide, Neuromedin B (NMB), is a component in the innate immune response to Influenza A viruses in mammalian in vitro and in vivo models. A549 cells lacking the NMB receptor were more susceptible to H1N1/PR8 infection [72]. Our study implicates a role of NMU in the innate immune response. Human fibroblasts with an active NMU pathway were more susceptible to WNV-Kun infection. It is likely that suppression of insulin secretion during WNV infection in human cell culture prevented activation of JAK/STAT innate antiviral immune pathways, leading to a weakened response to infection.

Mosquito orthologs for Lst have been identified in *Ae. aegypti* and *An. gambiae* [73]. The investigation of mosquito Lst in a future study could determine if a mosquito decretin hormone would activate higher *ilp* expression to suppress viral infection through increased activation of antiviral pathways. In human innate immunity, it is possible that disruption of the NMU pathway would lead to protection from WNV infection in pancreatic β-cells and other cell types, as well as other mammalian models. A known antagonist of NMU signaling has been identified [74]. However, disruption of NMU activity may have adverse side effects. NMU knockout mice show hedonic eating, preference for high-fat diets, and increased levels of obesity [31]. The role of NMU as pro-viral during WNV infection and in mammalian immunity should be studied further, specifically how it signals during virus infection and acts a suppressor of insulin secretion and the insulin-mediated antiviral response. Examining the roles of insulin during WNV infection will have a direct impact on improving therapeutics for infected individuals. Taken together, the work presented here uncovers immune signaling nodes that could be targeted to reduce overall viral load in vectors, thereby preventing transmission between mosquito and human populations.

## Figures and Tables

**Table 1:** Statistics of individual infection trials.

Experiment #	Hazard Ratio (*Lst^MI06290^*/*y^1^w^1^*)	*p*-Value
1	0.3181	0.0002
2	0.1297	<0.0001
3	0.3570	0.0026

## Data Availability

The raw data supporting the conclusions of this article will be made available by the authors upon request.

## Supplementary Materials

The following supporting information can be downloaded at: https://www.mdpi.com/article/10.3390/insects15060446/s1, Figure S1: Original uncropped western blot images for Figure 1 and Figure 5.
