# Supplementary figures and images for: Drosophila melanogaster Limostatin and Its Human Ortholog Promote West Nile Virus Infection
Source: Insects. 2024 Jun 12;15(6):446. doi: 10.3390/insects15060446 (PMC11203814; doi:10.3390/insects15060446)

Anti-phospho-Akt

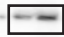

Figure S1

Anti-Akt

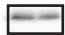

Anti-actin

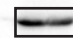

Figure S5

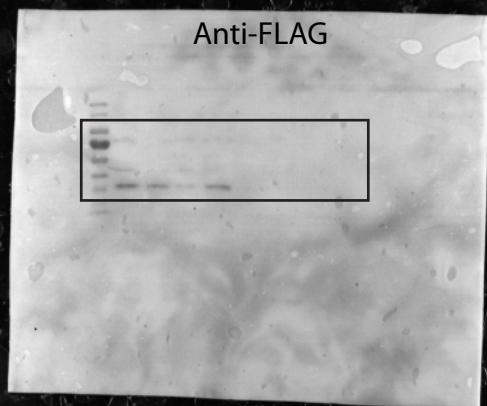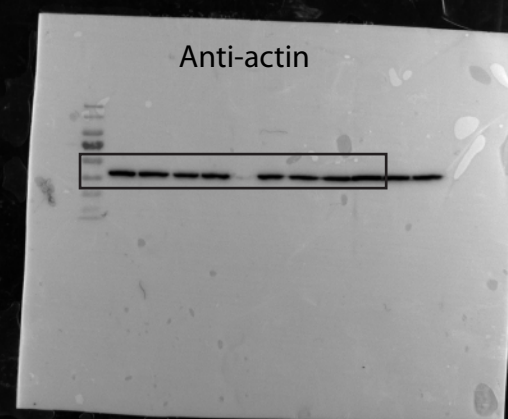

Supplement: Supplementary file 1 [file insects-15-00446-s001.zip › insects-3015604-supplementary.pdf]
